# Participation and Organizational Commitment in the Mondragon Group

**DOI:** 10.3389/fpsyg.2022.806442

**Published:** 2022-03-15

**Authors:** Alfonso Rodríguez-Oramas, Ana Burgues-Freitas, Mar Joanpere, Ramón Flecha

**Affiliations:** ^1^Department of Sociology, University of Barcelona, Barcelona, Spain; ^2^Department of Sociology, University of Granada, Granada, Spain; ^3^Department of Business Management, Rovira i Virgili University, Tarragona, Spain

**Keywords:** Mondragon Corporation, worker cooperatives, organizational commitment, participation, psychological wellbeing, organizational democracy

## Abstract

The scientific literature has shown Mondragon Corporation (MC), with 65 years of history, as a clear example that cooperativism can be highly competitive in the capitalist market while being highly egalitarian and democratic. This cooperative group has focused on its corporate values of cooperation, participation, social responsibility, and innovation. Previous scientific research reports its enormous transformative and emancipatory potential. However, studies on the effects of various types of worker participation on competitiveness and workers’ psychological wellbeing in this cooperative group exist to a lesser extent. Specifically, one aspect that needs further empirical research and that represents a competitive advantage for Mondragon is the degree of commitment and emotional attachment that can be observed in the people who work there. For this reason, this article aims to identify key elements of the democratic participation of workers in these cooperatives that relate to the development of organizational commitment. Based on a communicative and qualitative approach, data collection included 29 interviews to different profiles of workers (senior and junior workers, members and non-members of the cooperative, and researchers involved in the cooperatives) from eight different cooperatives of the Corporation. Through this research methodology, the participants interpret their reality through egalitarian and intersubjective dialogue because their voices are considered essential to measure the social impact. This study found three different ways in which the democratic participation of worker-members in management and ownership contributes to developing affective organizational commitment among those working in Mondragon cooperatives, generating positive psychological and economic outcomes for both workers and cooperatives.

## Introduction

Worker cooperatives represent a democratic alternative to the traditional mainstream model of creating value and organizing work. In them, workers are highly involved in labor and capital, mainly through shared equity and participation in decision making at all levels (Cheney et al., 2014). According to the literature, cooperatives and worker-owned enterprises that combine employee involvement and ownership can match or even exceed the productivity of conventional enterprises (Bradley et al., 1990; Bartlett et al., 1992; Doucouliagos, 1995; Logue and Yates, 2006; Artz and Kim, 2011). At the same time, cooperatives provide greater job security, particularly during times of crisis (Birchall and Ketilson, 2009), often report higher pay and benefits relative to conventional firms when they succeed in a particular industry (Artz and Kim, 2011), and contribute to dignify working life and reduce poverty (Logue and Yates, 2006). Furthermore, worker cooperatives can create some stress and demand specific emotional labor due to the complexities of their democratic organization. But compared to conventional firms, they usually offer greater emotional freedom and a more comprehensive range of genuine emotional displays (Hoffmann, 2016). For these and other reasons, cooperatives are broadly considered a factor promoting their communities’ economic and social development (Johnstone and Lionais, 2004; Erdal, 2011; United Nations General Assembly, 2017).

The Mondragon Corporation (MC) is the largest and most successful group of cooperatives globally (Wright, 2010; International Co-operative Alliance, 2012), a clear example that worker cooperatives can be competitive in the capitalist market, egalitarian, and democratic. The group comprises more than 200 firms, 96 cooperatives and employs more than 81,000 people worldwide (Mondragon Corporation, 2021). MC is the leading business group in the Basque Country, where its headquarters are located, and one of the largest in Spain, having sales in more than 150 countries. In 2003, Fortune magazine ranked one of the best places to work in Europe due to the working conditions, absence of hierarchical atmosphere, equality, and personal decision-making capacity, among other aspects. Unlike most worker cooperatives, which are rare in capital-intensive sectors such as domestic appliances and industrial machinery (Dow, 2003), the Mondragon cooperatives have a presence in the finance, industry, retail, and knowledge sectors (Mondragon Corporation, 2020). Furthermore, MC has successfully managed to overcome economic recessions and internal challenges while continually expanding since its inception back in 1956, remaining committed to democratic work practices and dedication to local communities where they have a presence (Cheney et al., 2014).

The characteristics of the democratic model of MC and its relation to its economic and social success have long interested the scientific community (e.g., see Meek and Woodworth, 1990; Moye, 1993; Taylor, 1994; Whyte, 1995; Cheney, 2002; Mintzberg, 2009; Campbell, 2011; Redondo et al., 2011). However, one aspect that needs to be explored in more depth is the impact of the democratic participation of MC worker-members on their affective organizational commitment (Allen and Meyer, 1996), a factor that may represent a competitive advantage for the cooperatives (Agirre et al., 2014). Previous research has pointed to generalized satisfaction among worker-members of MC, particularly among highly qualified professionals who could earn more in traditional mainstream companies but who instead decide to remain in Mondragon (Flecha and Santa Cruz, 2011). Consistent with this assertion, there is evidence showing how the organizational commitment of MC workers may be caused by the democratic management model implemented in those cooperatives (Agirre et al., 2014). This management model relies on decentralizing and distributing power, promoting employee participation in decision-making, and implementing a quality-based management based on cooperative principles. Similarly, studies have found that organizational democracy positively impacts value-based commitment (Weber et al., 2020). Besides, workers are less likely to leave their companies due to job demands in worker cooperatives than in traditional mainstream companies (Park, 2018). However, more empirical research is still needed to fully understand how the democratic participation and involvement in the Mondragon cooperatives may lead to enhanced organizational commitment of their workers.

This article aims to contribute to fill this gap in the literature by presenting the results of a qualitative study carried out in MC cooperatives, which focused on identifying ways in which the democratic participation of workers in management and ownership influences the development of affective organizational commitment. The evidence presented suggests that participation in management increases the identification of worker-members with the governance of the cooperative (i.e., the democratically elected members of the governing bodies and the leadership of the company) and promotes involvement of the worker-members in decision-making at the highest level which increases their understanding of the business and empathy toward the management. Participation in ownership appears to generate emotional attachment to the cooperatives as worker acknowledge themselves as co-owners of the cooperatives and part of a collective business effort. In sum, participation in management and ownership appears to generate identification with, involvement in, and emotional attachment of workers to their cooperatives, all of which are elements that relate to the definition of affective organizational commitment (Allen and Meyer, 1996).

In the following, the article begins with a brief review of scientific literature on the relationship between organizational commitment and organizational democracy is reviewed. Then, the types of participation and involvement of workers that exist in MC cooperatives are contextualized. Third, the methodology followed is presented in detail. Fourth, the study’s main findings are presented in three main sections. Finally, conclusions are presented drawn from the findings, as well as a critical discussion about them.

## Organizational Commitment and Organizational Democracy

The leading concept of organizational commitment, which emerged three decades ago as a construct related to the study of work attitudes and behavior, is defined as “a psychological link between the employee and his or her organization that makes it less likely that the employee will voluntarily leave the organization” (Allen and Meyer, 1996). According to this conceptual model, the psychological linkage between employees and their organizations can take three distinct forms (Meyer and Allen, 1991): *affective commitment*, which refers to identification with, involvement in, and emotional attachment to the organization; *continuance commitment*, which refers to commitment based on the employee’s recognition of the costs associated with leaving the organization; and *normative commitment*, which refers to commitment based on a sense of obligation to the organization. In other words, employees may decide to remain with an organization because they want to (affective), because they have to (continuance), or because they ought to (normative) (Allen and Meyer, 1996).

While all three forms of organizational commitment correlate negatively with withdrawal cognition and turnover, they differ in the way they correlate with organization-relevant and employee-relevant outcomes: affective commitment has been found to have the strongest positive correlation with attendance, job performance, and organizational citizenship behavior, and to be negatively correlated with stress and work-family conflict; besides, affective commitment correlates strongly with job involvement, occupational commitment, and, most vital of all, job satisfaction (Meyer et al., 2002). Thus, of the three forms of organizational commitment, affective commitment has the most positive consequences for employees, just as it does for employers (Meyer and Maltin, 2010). From the point of view of the firms, affective commitment represents a competitive advantage. When employees want to remain in the organization and believe it is the right thing to do, they tend to be happier, more satisfied, more self-directed, healthier, more engaged, and more willing to exert discretionary effort on behalf of the organization (Meyer et al., 2012).

In the last decade, a large body of research has widely studied the correlation and interaction of organizational commitment with numerous psychological variables in conventional organizations and capitalist firms (e.g., Morin et al., 2011; Farooq et al., 2014; Choi et al., 2015; Kim et al., 2016; Meyer et al., 2018; Yao et al., 2019). However, democratic workplaces and democratic organizations have received far less attention on this matter. Notable exceptions are the studies that have analyzed the relationship between organizational democracy and different psychological and behavioral outcomes, including affective and normative commitment (e.g., Weber et al., 2009, 2020; Unterrainer et al., 2011). The term *organizational democracy* is referred to as an on-going, broad-based, and institutionalized employee participation that is not *ad hoc* or occasional in nature (Weber et al., 2009). In a recent meta-analysis of psychological research on organizational democracy, Weber et al. (2020) used three indicators of this construct: (1) *Structurally anchored employee participation in organizational decisions* (SAEP), focused on the organizational decision structure, distinguishing among several types of enterprises according to a typology that ranges from hierarchically structured enterprises to self-governed employee-owned firms and worker cooperatives; (2) *Employee participation in collective ownership* (EO), focused on the property situation, encompassing two variants: whether an employee possesses ownership shares of the company or not, and the number of his/her ownership shares; (3) *Individually perceived participation in organizational decision making* (IPD), focused on the participation of individual employees, reflecting the degree of employees’ *actual* and *direct* participation in strategic or tactical decision making as perceived by themselves. According to their findings, value-based commitment [an aggregate of affective and normative commitment derived from Meyer et al. (2006)] is positively influenced by all three indicators of organizational democracy almost to the same extent. Additionally, the authors found that IPD partially mediated the positive effects of SAEP and EO on value-based commitment, showing the specific relevance of workers’ direct involvement in strategic and tactical decisions for promoting positive forms of organizational commitment.

Only a handful of studies have analyzed aspects related to organizational commitment in worker cooperatives. However, none have analyzed the relationship between the democratic participation of worker-members and the development of organizational commitment. A study of French agricultural cooperatives concluded that members participate in the governance of their cooperative when they are attached to it affectively and trust the directors (Barraud-Didier et al., 2012). Another study in cooperatives in Ecuador found that certain variables related to job position such as higher salary levels, involvement in strategic management (specifically being an executive), or temporary contracts all had a significant and positive relationship with organizational commitment (Hidalgo-Fernández et al., 2020). In a comparative analysis between worker cooperatives and capitalist firms in South Korea, Park (2018) found that the increase in job demands and workloads in cooperatives did not seem to reduce the organizational commitment of their members, most likely because they are provided with greater autonomy, organizational support, decision-making participation, and social support as compared to their capitalist counterparts. The only study on MC cooperatives is that of Agirre et al. (2014), which found that decentralization and participative decision making, both critical components of Mondragon’s management model, have a significant influence on worker’s organizational commitment. Additionally, the authors found that organizational commitment in MC cooperatives has a positive albeit indirect influence on business performance through its impact on the market orientation of the cooperatives (i.e., the generation and distribution of market information and subsequent response to it).

In retrospect, research on organizational commitment has made significant contributions in areas such as behavioral sciences and organization studies. The universal character of the construct allows the replication of quantitative research methods in various types of organizations, as is the case of the studies on worker cooperatives described above. To strengthen this fruitful area of investigation, researchers could complement the existing quantitative studies with qualitative analyzes that allow them to recover the voices and interpretations of the investigated subjects. This would contribute to broadening scientific knowledge about the processes that underlie the development of organizational commitment in organizations and its interaction with diverse psychological and social variables.

## Workers’ Participation in Mondragon Corporation Cooperatives

### Participation in Management

The worker cooperatives are the base level of organization in MC. In them, workers have multiple opportunities to extensively participate in decision-making processes of issues that are transcendental to the cooperatives’ trajectory and their own work life. The democratic participation in the management of worker-members takes place through specific governing bodies shared by all Mondragon cooperatives (see Forcadell, 2005, p. 260–261). At the top, composed of all worker-members, the General Assembly is the supreme authority of the cooperative, which gathers at least once per year to approve strategic plans, nominate the Governing Council, the Audit Committee, and the Social Council. Every member of the cooperative can vote in the General Assembly, and their votes are weighted equally. The Governing Council is a group of up to twelve worker-members elected for 4-year periods. It is in charge of presenting the annual operational plan for approval to the General Assembly, nominating the managers of the cooperative, and periodically monitoring their performance to assure that the company’s management complies with the directives of the General Assembly. The Audit Committee audits all the accounts presented to the General Assembly and obtains supplementary information when required by the cooperative members.

The governing bodies mentioned above assure workers’ participation in management in the form of control instead of directly making management decisions (Campbell, 2011), which are the responsibility of the Manager and the Management Committee, a consultative body of managers of the cooperative. Along with them, another body called Social Council represents the worker-members before the authorities of the cooperative, having an advisory character to the Governing Council, and acts as a labor union and a communication channel between management and workers (Forcadell, 2005). Their co-workers directly elect the representatives of the Social Council, and the issues that they address include working conditions, compensations, remunerations, social security, and many other social topics (Campbell, 2011). Additionally, each member of the Social Council organizes individual meetings with the workers that they represent, called Small Councils, where all the issues mentioned above are discussed in-depth (Altuna, 2008).

### Participation in Ownership

As worker-owned enterprises, the MC cooperatives are characterized by high levels of participation of their workers in decision-making processes and equity through individual participation of their members in ownership. Once they are eligible for membership and contribute their respective share of capital to the firm, workers acquire a series of obligations toward the cooperative, including participating in democratic processes of the cooperative and taking part in economic losses when applicable. In addition, they obtain rights such as participating and voting in decision-making processes, being eligible to become part of governing bodies, and participating in the cooperative’s profits. Of any given MC cooperative, not more than 20% of its workforce are non-member workers, and temporary contracts cannot last more than 5 years, as set by local regulations for worker cooperatives in the Basque Country (Altuna, 2008; Flecha and Santa Cruz, 2011).

In recent years CM has carried out different actions to replicate its successful cooperative model abroad. It includes the creation of mixed cooperatives and the implementation in its subsidiaries of its Corporate Management Model to promote the dissemination of democratic organization practices and participatory management (Flecha and Ngai, 2014), or the collaboration between MC and the United Steelworkers Union in the United States to develop a union co-op model based on the values of worker ownership, collective organization, democracy, and solidarity (Schlachter, 2017).

### Tensions and Contradictions in a Context of International Growth

The extent to which MC cooperatives maintain their cooperative values and assure workers’ participation as they grow and compete in international markets has been the subject of discussion in the past decades. A good part of the analyses has been framed within the *degeneration vs. regeneration* debate (Cornforth, 1995). The degeneration thesis (Webb and Webb, 1921; Meister, 1984) states that to survive worker cooperatives gradually and inevitably adopt organizational practices and priorities that erode the democratic participation of workers in decision making, thus becoming increasingly capitalist. In contrast, several authors that criticize such a deterministic stance state that worker cooperatives can maintain their original nature in the long-term, since worker-members can set up regeneration processes that contribute to restoring democratic, participative, and social functioning (Batstone, 1983; Rosner, 1984; Hunt, 1992; Cornforth, 1995).

A number of recent studies have criticized degenerative processes that have eroded the democratic participation of workers in MC cooperatives, although in some cases also acknowledging efforts in the opposite direction. For instance, Heras-Saizarbitoria (2014) states that Mondragon’s basic cooperative values such as democratic organization, participatory management and education have become decoupled from worker’s daily activity, and that the principle of secure membership and employment is the only one that encourages “most workers to remain quiet and compliant in a system that gives them limited ways to participate.” On a qualitative study of Fagor Ederlan, an MC multinational worker cooperative, Bretos et al. (2018) found that international growth contributed to lessen worker-members participation in favor of managerial control. Similarly, during its expansion period, Storey et al. (2014) found that Eroski, MC largest retail cooperative, experienced a sharp reduction of its percentage of members, gave greater priority to its economic goals, and suffered from passivity and loss of interest by its members. However, the authors also assert that the economic crisis in the post-2008 period played an essential role in the re-emergence of cooperative practices in the cooperative, as members showed an increasing desire to be informed, attend meetings and actively take part in decision-making committees and governing bodies. In a study of Fagor Ederlan, Fagor Electrodomésticos, and Maier Group, Bretos et al. (2020) state that those cooperatives have developed interesting regeneration initiatives such as cooperativizing some of their domestic subsidiaries, updating and institutionalizing cooperative education and training for managers and members of the governing bodies, and revitalizing certain employee voice structures. However, the authors emphasize that the cooperatives have kept growing through the acquisition of capitalist firms. Participation in their work areas continues to be shaped by dominant, managerially controlled systems, and education and training offered to rank-and-file worker-members suffer from a systematic deficit. It suggests that regeneration and degeneration are not mutually exclusive and can occur simultaneously.

Transferring the democratic participation of workers in management and ownership to subsidiaries of MC cooperatives has also been the subject of debate. While MC has been criticized for prioritizing the transfer of shallow, managerially oriented forms of workers’ participation (Bretos and Errasti, 2017), research also suggests that some worker cooperatives have taken interesting steps toward “cooperativizing” their subsidiaries. They include the transformation of Eroski hypermarkets into cooperatives (Arando et al., 2015), the transformation into mixed cooperatives of some subsidiaries of Maier Group (Flecha and Ngai, 2014), the creation of a mixed cooperative subsidiary by Copreci, or the integration as production plans of Fagor Ederlan’s subsidiaries Automoción and Victorio Luzuriaga Usurbil with the inclusion of their workers as members of the parent cooperative (Bretos et al., 2020). However, according to research MC cooperatives have been less successful in transferring their cooperative model to their subsidiaries abroad. Bretos et al. (2019) state that Fagor Ederlan and Fagor Electrodomésticos became “capitalist” firms composed of a cooperative headquarter in their multinational expansion and a capitalist periphery of subsidiaries in which cooperative membership rights are restricted for workers.

Similarly, in an analysis of Mondragon’s Chinese factories, Errasti (2015) suggests an apparent disconnect between the organization’s discourse regarding the encouragement of worker participation in subsidiaries and the practices observed in the Kunshan Industrial Park and concludes by stating that its Chinese subsidiaries do not differ significantly from traditional foreign subsidiaries. In this regard, Flecha and Ngai (2014) suggest that economic aspects, legal difficulties in the destination countries, the lack of a cooperative culture, and a desire to protect investments hinder MC from effectively expanding its cooperative model to its international subsidiaries. Nevertheless, the authors acknowledge that the actions taken by some cooperatives (specifically Maier, ULMA Construction and RPK, a former MC cooperative) to implement a corporate management model in their subsidiaries, albeit limited to one concrete dimension, are valuable first steps toward the objective of expanding the cooperative culture abroad.

## Materials and Methods

### Research Question and Context of the Study

The present study aims to address the following research question: How does the democratic participation of worker-members in management and ownership influence the development of affective organizational commitment in MC cooperatives?

The results presented here build on the research “The Contribution of Competitive Cooperativism to Overcoming Current Economic Problems” (Cooperativismo Competitivo: Aportacions a la sostenibilidad y calidad del empleo en el momento económico actual) (CREA, 2012–2014), funded by the Spanish Ministry of Science and Innovation through the National R&D Program. The two main objectives of the R&D project were, first, to identify successful practices developed by worker cooperatives that improve the quality of work, particularly of the groups that suffer greater vulnerability; and second, to identify the possibilities of transferring those successful cooperative actions to other companies and other territories. For that purpose, numerous case studies of Spanish worker cooperatives were carried out, as well as four cross-sectional studies to identify and analyze elements that have a positive impact on the quality of work and life of different groups such as: women, young people, people with low qualifications and people with disabilities.

The study presented below corresponds to a subset of the project’s field work. Specifically, 16 interviews corresponding to two in-depth case studies of the Alecop and Maier cooperatives are used in this study. In addition, with the aim of achieving a better understanding of the matter, the field work was complemented with 13 interviews of workers from six additional MC cooperatives: Fagor Automation, Laboral Kutxa Mondragon University, Orona, ULMA Architectural Solutions-Polymers, and ULMA Group. Table 1 shows the distribution of interviews carried out for this study.

**TABLE 1:** Distribution of communicative interviews.

Sector	Cooperative	Participants
Finance	Laboral Kutxa	1
Industry	Fagor Automation	2
	Maier	7
	Orona	1
	ULMA Architectural Solutions–Polymers	1
	ULMA Group	2
Knowledge	Alecop	9
	Mondragon University	6
Total:		29

### Data Collection and Analysis

This study was carried out using a qualitative methodology, which allows delving into routines, problematic moments and the meaning in the lives of individuals (Denzin and Lincoln, 2011). Specifically, the research was developed using the communicative methodology (Gomez, 2021), which aims not only to describe social phenomena but also to provide scientific evidence of how social realities can be changed. In this methodology, researchers establish an egalitarian dialogue with the people investigated throughout the research process, searching for a common understanding of the reality based on validity claims. During the interviews, researchers bring to the dialogue the scientific evidence on the subject in question while the participants contribute with their daily life knowledge and experience. The joint interpretation of reality on an egalitarian level allows the identification of relevant topics that would not otherwise emerge in the research, and the active participation of the people whose reality is being studied increases the social impact of such research (Gómez et al., 2019). In the field of psychological research, this methodology has proven useful for generating scientific knowledge with substantial potential to improve peoples’ lives (Redondo-Sama et al., 2020).

In this study, to ensure a broader understanding of the reality, it was sought that the participants of the study had different profiles that allowed achieving a better interpretation of the situation in their cooperatives. The diversity of participants contributes to overcome the biased view of reality that some previous academic studies have offered when studying MC cooperatives derived from unilateral visions of the phenomenon (Morlà-Folch et al., 2021). In our study, profiles of interviewees included senior and junior workers, worker-members and non-members, management staff and members of the governing bodies, and researchers involved in the university of the corporation. Specifically, Table 2 details the profile of the people interviewed.

**TABLE 2:** Characteristics of interviewees.

	Description	Participants
Gender	Male	16
	Female	13
Position	President of the cooperative	1
	Manager of the cooperative	1
	Former Manager of the cooperative	1
	Human Resources Manager	1
	Member of the Governing Council	3
	Member of the Social Council	2
	Worker-member	9
	Worker-member, researcher	3
	Worker-member, student	4
	Worker, not member	4
Total:		29

Regarding the two in-depth study cases carried out in Alecop and Maier, first, an informant in each cooperative was contacted to access the interviewees. In the first conversation, the research and its objectives were explained. Then, the contacts of people who might be interested in conducting the interviews were facilitated. Regarding the complementary interviews, a snowball sampling was carried out, in which informants who were not designated by the cooperatives facilitated the contact of various workers of MC cooperatives.

The interviews followed a semi-structured protocol with themes that emerged from the literature review designed to explore the relationship between the democratic participation of worker-members in management and ownership with the development of organizational commitment in the cooperatives. During the dialogue, the researcher presented the scientific evidence available, and the interviewees contributed their points of view on those investigations from their daily life knowledge. The conversations were open and allowed the interviewees to expand on the topics they wanted, offering time to discuss those issues that they considered most relevant, some of which were not considered initially. The interviews took place at the site the interviewees decided on to ensure comfort with the conversation. For this reason, some interviews took place in the workspaces of the cooperatives, while others took place in other spaces suggested by the interviewees.

The interviews were conducted between 2012 and 2014. The average duration of the interviews was approximately 1 h, but in no case did the interviewer limit the time of the interview. All were recorded and transcribed verbatim for their analysis. All participants involved in the study provided either verbal or written consent agreeing to participate in the research. They were also informed about their right to leave the research and remove their data from the analysis at any time if they wanted. The study was approved by the Ethics Committee of the Community of Research on Excellence for All (CREA) with the number 20211031.

For the analysis of the interviews, a first screening was carried out. All quotations that indicated a relationship between democratic participation of workers in MC cooperatives (i.e., participation in management and participation in ownership) were identified. In a second step, those quotations were classified following the next analysis chart (see Table 3). Based on the communicative methodology, the chart differentiates between transformative and exclusionary dimensions of three categories of analysis.

**TABLE 3:** Analysis chart.

Categories of analysis	Exclusionary dimension	Transformative dimension
Emotional attachment to the cooperative	1	2
Identification with the cooperative	3	4
Involvement in the cooperative	5	6

The results of the analysis were then classified into three final categories:

• *Identification with the democratic governance of the cooperative*, containing evidence of grassroot democracy underlying the process of election of members for the governing bodies.
• *Involvement in management that enhances understanding of the business*, containing evidence of learning processes that take place as worker-members participate in the governing bodies.
• *Emotional attachment to a co-owned business effort*, containing evidence of enhanced motivation and engagement of workers that acknowledge themselves as co-owners of the company.

The quotations presented in the following section represent the findings that had consensus among the interviewees. No major differences in the basic interpretation of the phenomenon were identified. However, some differences between the answers of worker-members and temporary workers are specified. Only in some specific cases, the interpretations varied, which was specified in the description of the results.

## Results

Our data analysis suggests that the democratic participation of worker-members in MC cooperatives (in management and ownership) contributes to developing affective organizational commitment in at least three ways. First, worker-members feel identified with the cooperative’s governance since they postulate and democratically elect the colleagues they trust to be part of the governing bodies. Second, as worker-members elected for the governing bodies are involved in the most important decision-making processes of the cooperative, they must receive intensive training on the operation of the cooperative and interact with the management staff, which significantly enhances their understanding of the business. Third, worker-members become emotionally attached to their firms as they acknowledge themselves as co-owners of the cooperatives and part of a collective business effort.

### Identification With the Democratic Governance of the Cooperative

The democratic functioning shared by all MC cooperatives enables worker-members to participate in transcendental decisions for the cooperative, such as the election of the company’s leadership. During the General Assembly, all members vote to choose the president of the cooperative, who simultaneously presides the Governing and Social Councils. The Governing Council postulates the candidates. The General Assembly has the power to elect one or reject the nominees if they consider that none is adequate to preside the cooperative. Through this combination of representative democracy (postulation of nominees by the Governing Council) and participatory democracy (voting at the General Assembly), worker-members have a say in the appointment of the leadership:

“Normally, we in the Governing Council nominate those who we believe that may like the company, and that we like them [their profile]. And you say, “Ok, it seems to me that he is a good guy.” You can nominate 2 or 3 people and then the [General] Assembly decides which one. Or they can censure, and they don’t choose any of them. That has happened in [Fagor] Industrial” (*Female; worker-member; Fagor Automation*).

As expressed by this former member of the Governing Council in Fagor Automation, the presidency of the cooperative is not given for granted beforehand the General Assembly takes place. Instead, worker-members have the last word on who may preside over the company. Similarly, the management of the cooperative must count with and maintain the trust of the worker-members, although the process of choosing and removing when applicable operates indirectly through the action of the Governing Council:

The manager is elected by the Governing Council, which is the representative of the cooperative. They elect the manager for 5 years, although they can dismiss him/her at any time because it is a position of trust. In this specific case [of our cooperative], this person has been in the job for 10 years now, he was trained in the cooperative, he entered young and climbed positions, and now he is the general manager (*Male; worker-member; Laboral Kutxa*).

As explained above, the general management of the cooperative is considered a position of trust, which means that managers can be removed at any time if worker-members, through their representation in the Governing Council, assess that the direction of the cooperative is not the right one. However, as he also explains, general managers can be re-elected for additional periods if the overall results are considered positive for the cooperative.

It is worth noting that some of the interviewees coincided in pointing out that such trust in the leadership, along with other values such as transparency and honesty, may motivate the worker-members. The following quote showcases this idea:

The important thing in a company is that people who go to work motivated. (…) So, three key elements of motivation are: transparency, promotion of people, and honesty and credibility. That means that the people who are leading the company are trustworthy, that the managers are trustworthy, that they are honest. In other words, to create a climate of motivation. (…) The advantage of cooperatives is that a person feels better paid within the cooperatives, although they are never satisfied, [and] they feel they participate in the management, because they have transparency and they have information, and they feel that those who manage the cooperative are honest, they are close, and generally feel that they are honest. There will always be criticism, but I think there is honesty and credibility, and that is a bit of the great advantage of the cooperative (Male; former Manager of the cooperative; MAIER).

As seen in the previous quote, this former Manager of Maier emphasized that the perception of having honest and trustworthy leaders is vital for creating a climate of motivation in any company, be it cooperative or capitalist. Furthermore, he asserted that in worker cooperatives, worker-members might perceive the leadership as being more honest, transparent, and close to the workforce than other types of firms.

In that sense, according to the dialogues with the interviewees, it appears that the democratic process through which representatives of the governing bodies and the leadership of the cooperative are elected is a key element that contributes to develop trust and identification with the governance of the cooperative: “*There are advantages in giving a vote to the worker-members of the cooperative because it generates greater commitment in those elected [to form part of the governing bodies] and people identify themselves more*” (*Male; worker-member; ULMA Group*). As suggested by this worker-member of ULMA Group, this process appears to have a double positive effect. On the one hand, it influences the identification of worker-members with the governance of their cooperatives. Such identification appears to be based on trust, as worker-members themselves nominate and choose the colleagues who they consider most suitable for the positions. On the other hand, being designated by their colleagues for those representative positions potentially increases the commitment of the elected members, as they are backed by their peers throughout the process.

Reinforcing the previous idea, some testimonies from interviewees suggest that the grassroot democracy that underlies the election of members for the governing bodies contributes to their identification with the governance of the cooperative. Specifically, because it assures that people in whom they trust make the most important decisions in the cooperative. This was clearly stated by a college professor and worker-member of the Mondragon University cooperative:

“It is important that people choose. I mean, we choose among the people those that we see in that position. Then we make a proposal, ‘Hey, we would like Pedro.’ I think that’s good, that in the representation be someone who is trusted. The confidence of saying, ‘Look, we trust you and you are in a position where the strategic decisions [of the cooperative] are made.’ I think it gives you more peace of mind. I think so” (*Female; worker-member; Mondragon University*).

As showed in the previous quote, the decision to postulate someone to the governing bodies is usually based on recognizing the commitment of a member toward the cooperative and his or her potential to look after the interests of both the business and the workers. In this process, worker-members rarely nominate themselves. Instead, worker-members postulate other colleagues they consider trustworthy and capable of achieving good results in the crucial tasks they will be assigned.

In sum, the evidence presented above suggests that the democratic election of representatives for the governing bodies may increase the identification of worker-members with the governance of their cooperatives, i.e., with the members elected for those bodies and, at least to some extent, with the leadership of their companies.

### Involvement in Management That Enhances Understanding of the Business

The democratic process described in the previous section opens the possibility for any worker-member of the cooperative to be potentially elected for the governing bodies despite his or her job position or academic qualifications. Often, the members who join the governing bodies, particularly the Governing Council, lack the basic training required to understand the information at their disposal and make informed decisions. This limitation was clearly explained by this member of the Social Council at ULMA Architectural Solutions-Polymers:

Normally, the Governing Council is not all engineers or businessmen. There are people who work on the production line, people who have medium training, or even people who have high training. but in the end they are not in contact [with the administration]. For example, there may be people who are not in direct contact with the market and do not have that global perspective of the entire company to say, “this is wrong” or “this is good.” It is not usually a common thing (*Female; member of the Social Council; ULMA Architectural Solutions-Polymers*).

As described above, all worker-members, including those that work on production lines and equivalent positions, are eligible for the Governing Council. Moreover, according to the interviewees, this situation is relatively common. In some cases, like in Alecop, a percentage of seats in the Governing Council (and other governing bodies) is even reserved for university students who work part-time at the cooperative. This situation represents a challenge for all MC cooperatives. They are faced with the need to provide intensive specialized training on cooperative business management to members that join the Governing Council with a wide range of profiles. While the specificities of the training may vary from one cooperative to another, they usually include some type of regulated training on technical matters like the interpretation balances and result accounts, among many other topics, as explained in the following quote of a member of the Governing Council of Maier:

“That training (…) is a challenge, a huge challenge to provide that. In the end, any member is eligible to the Governing Council, anybody, regardless of their education, and that person will have to make decisions once he/she is there. So, the regulated training is the interpretation of income statements and their values, although that isn’t enough, no. [However] you learn during those 4 years in the Council so much! Of course, you have meetings that are only for the Governing Council members, and then you also have meetings with the entire Directorate, the Managing Director, the Human Resources Department. You learn about your own cooperative, the systems, the processes, how it works and how things are done” (*Male; member of the Governing Council; MAIER*).

However, as the previous quote explains, only part of the learning comes from formal training. During the years in the Council, the members learn a great deal from the direct interaction with the entire management staff of the cooperative, which may include the managing director, the finance department, the human resources department, and others. Those interactions are equally important, if not more, in helping the worker-members of the Governing Council to grasp a deeper understanding of the status of the cooperative and the business.

More importantly, joining the Governing Council entails being involved in transcendental decision-making processes for the cooperative such as elaborating the annual operational plan, nominating the management staff, and monitoring their performance. Often, the members’ decisions are tough to make, as when cooperatives have needed to find ways of preserving employment at the expense of individual gains during the economic recession. Other times decisions may imply restructuring the cooperative or internationalizing its operation to compete globally. In either case, according to the interviewees, the direct involvement of worker-members in decision-making and management through their participation in the Governing Council radically transforms their understanding of the cooperative and makes them more emphatic with those in charge of managing the company. This idea was expressed by a college professor and worker-member at Mondragon University as follows:

“The experience you acquire helps a lot in the training of workers because you see how the company works. That a person who works on a machine has the possibility of being on the governing council and making decisions. that person when he returns to the machine works in a different way. Because he already knows where the company is going and why those decisions have been made, because you are the manager. And you are going to have to make some decisions that not everybody may like. The next time that you aren’t in the governing council, but those same decisions are made, you’ll also understand, ‘Well, I had to make those decisions too.’ Even if you are part of the Council only 4 years, that lasts forever” (*Female; worker-member, researcher; Mondragon University*).

Furthermore, as the interviewee emphasizes, the experience of forming part of the Governing Council has a long-lasting impact on the motivation and commitment of worker-members once they reincorporate to their job positions at their cooperatives, since they gain a perspective of the business that they could not have gained otherwise.

Similarly, according to the interviewees’ testimonies, the experience of forming part of the Social Council contributes to increasing the business’s understanding of those elected for this representative body. Among other responsibilities, the members of the Social Council are responsible for communicating to their colleagues the status of the cooperative, often through monthly gatherings called Small Councils. The information is provided directly by the management of the cooperative and the Governing Council. It may include financial balances, economic forecasts, or any other relevant cooperative updates. This was explained by two worker-members of Fagor Automation, one of whom previously had formed part of the Social Council:

*Female worker-member*: I understand now. When I see them [the current members of the Social Council] I understand them completely. We always say that each member should give those 4 years in the Social Council to value what it takes them to prepare the document they present every month. I used to prepare slides, “Here you have the economic results, the sales, the most important things that the manager says” every month.

*Male wormer member*: You also understand the balance sheets more.

*Female worker-member*: Yes, that is also positive. You learn and say, “Look, this is the operating income. Look how this affected that.” Or the financial manager may come and explain how the dollar affects the operation, because we have subsidiaries outside and there we sell in dollars. You learn those things (*Female and male; worker-members; Fagor Automation*).

As shown in the quote above, the effort that the members of the Social Council put on processing and communicating the information of the business to their colleagues impacts not only their understanding of the business, but also their empathy toward the members of the governing bodies of the cooperative.

In the case of the youngest participants of this study, namely the student worker-members of Alecop who work part-time at the cooperative as they conclude their university studies, their involvement in the governing bodies appears to have had a direct impact on their commitment to the cooperative. Specifically, when asked about her appreciation of the experience as a worker in Alecop, one of the interviewees expressed that she had learned far more about cooperative companies than at the university, since there she had the chance to experience first-hand the democratic functioning of a cooperative:

The experience is very enriching. (…) I wanted to get involved and I found it very enriching. Both as a cooperative member and as a worker, I mean, as a student that I am. I learnt how a Governing Council works, how you can deal with the manager, how the Management Committee or the Audit Committee work. Until you are inside you really understand how it works. And yes, you see that it really does work (…) I do value it very positively. I actually liked it. I would like to continue working here if it were up to me (*Female; worker-member, student; ALECOP*).

Students who work in Alecop are accepted as cooperative members for as long as their university studies last. Once their studies conclude, so does their status as members in Alecop. Only occasionally do the students continue working in the cooperative, and often they join other cooperative or capitalist firms in the region. Notably, the student above expressed her wish to continue working in the cooperative, derived from her fruitful experience participating in the governing bodies and her interaction with the management staff. While this experience cannot be extrapolated to all student worker-members, it does suggest that involving young and/or temporary workers in the management of the cooperative, for instance, through the governing bodies of the cooperative, may positively impact their affective organizational commitment.

### Emotional Attachment to a Co-owned Business Effort

While the evidence presented in the previous sections appears to suggest that democratic participation in the management of worker-members contributes to developing affective organizational commitment through somewhat indirect mechanisms, i.e., identification with the governance of the cooperative (trusting democratically elected representatives and leaders), and involvement in management that increases the understanding of the business (learning and developing empathy by participating in governing bodies), participation in ownership appears to contribute to the development of his type of organizational commitment in a rather straightforward way. When asked, most interviewees agreed on the idea that being a member of the cooperative, which first and foremost entails making an initial capital contribution, has a direct positive impact on the commitment that workers show in their workplaces and toward their cooperatives. This was stated as follows by a worker-member of ULMA Group with more than two decades of experience in the cooperative:

“You feel the company as yours. To become member, you must put money and that makes a big difference. You notice it with those who are not [members]. They don’t have that feeling. It makes you more committed to the cooperative” (*Male; worker-member; ULMA Group*).

As shown in the previous quote, workers develop an emotional attachment to the company as they become members and acknowledge themselves as co-owners. Furthermore, according to this and other testimonies, such feeling is not common among temporary workers, which is to be expected as they have not yet acquired cooperative membership.

Furthermore, testimonies collected show that worker-members are more engaged in their jobs and are more willing to work harder to achieve the objectives of the cooperative when they perceive that they have opportunities to participate, and their voices are listened to. In other words, it appears that the combination of ownership and democratic participation in management, and not the former alone, is crucial for developing the emotional attachment that leads to affective organizational commitment. This was explained by a worker-member of Maier as follows:

“Because, in the end, if you think that this is really something yours, and that your opinion counts, and that you are listened to and such, the level of involvement and the level of sacrifice that you are willing to assume for something that is yours is much higher than in anywhere else” (*Male; member of the Governing Council; Maier*).

The emotional attachment that interviewees showed in the dialogues was characterized by a recognition that co-ownership is accompanied by shared responsibility. The testimonies reflected an understanding of worker cooperative as a collective project in which both benefits and risks are shared by all members. Different examples given by the interviewees showed how under certain circumstances worker-members are will in to carry out actions that may affect their individual livelihood in the short term, with the objective of strengthening the cooperative in the middle or long-term. A worker-member of Alecop expressed this idea as follows:

“I believe that for those of us who are in cooperatives, apart from the salary, the cooperative offers you other things: security, [and] you participate in the company, for me that’s important. In other words, the day that I need to lower my salary, it will not be because someone else decided it, but because it is necessary. So, when we make money, we all win. And when we lose, we all lose. For me that’s a completely different feeling. You have responsibility, you have power and responsibility” (*Female; worker-member; Alecop*).

As seen above at times worker-members may even be willing to take actions such as decreasing their income or taking part of the losses because, as co-owners of the cooperative, they acknowledge their shared responsibility of seeking the best for the company. Similarly, as reflected in other testimonies collected in the study, in difficult times worker-members may be willing to put extra effort into their jobs when needed, even if it requires working extra hours. Not because anyone exploits them, but because they feel part of a collective project in which their individual contribution is important for achieving the collective goals of the cooperative. That feeling of belonging to a shared business effort appears to be a source of motivation, as expressed by a worker-member of the Mondragon University in the following quote:

“The other day I was talking to a friend, he is a trade unionist working in a traditional company. (…) He said that we worked more than 8 h because we wanted to. And we said that no, that here you have goals, and you have to achieve them, if it takes you 7 h as it takes you 10, that is your responsibility. (…) My job is to get this done, and that if 1 day I have to work 10 h, then the next day I work 7. But I do it. If I feel part of it. Well, I do feel part of this” (*Female; worker-member, researcher; Mondragon University*).

It is worth noting the difference in feelings that both workers have toward their companies, according to the interviewee’s testimony. The worker of the capitalist firm may never consider voluntarily working additional hours because his/her work would indeed benefit the owners but not him/herself. However, the interviewee feels part of the company because she effectively is, and for that reason, she sees no trouble in working extra hours whenever is needed. In other words, she perceives that all the effort put into this job will benefit herself and all the members of the cooperative.

Interestingly, participation in ownership may also impact temporary workers’ organizational commitment, albeit not in the form of an emotional attachment to the cooperative. Normally, after completing a 4-year trial period, temporary workers are invited to join as cooperative members if they perform adequately and embrace the cooperative values. According to our findings, the expectations of becoming cooperative members may play a role in motivating workers to be engaged in the cooperative and work hard in their jobs. A temporary worker of Orona explains this idea as follows:

“People like me with temporary contracts. The fact of becoming a member of a cooperative, like the one in which I am now, is an important step. In the end, becoming a member is better than a temporary contract in these times. You become member of a company that most likely won’t go bad, you know? They make you a member and you are part of the cooperative, they can’t kick you out. You are part of the company. So that means that during those 4 years people give everything and get involved in the company, to see if they make you a member and they consolidate you. All the people that I know that are my age. Most of them keep working hard after being consolidated as members” (*Male; worker not member; Orona*).

While the previous quote does not reflect emotional attachment to the cooperative, it does suggest that the influence of participation in ownership on organizational commitment may begin years before workers become members of the cooperative, as they engage in the cooperative culture and develop expectations of achieving a similar cooperative status.

## Discussion and Conclusion

The Mondragon Corporation (MC) has long interested researchers for being the most explicit example that cooperatives can thrive in capitalist markets while remaining fully committed to democratic values and work practices. One aspect that represents a competitive advantage for MC cooperatives is their worker-members generalized satisfaction and engagement, particularly noticeable among highly qualified professionals (Flecha and Santa Cruz, 2011). However, the relation between the democratic functioning of MC cooperatives and the organizational commitment of their workers has been studied only to a lesser extent. The present study contributes to fill that gap in the literature by investigating how the democratic participation of worker-members in management and ownership in MC cooperatives influences the development of affective organizational commitment (i.e., emotional attachment to, identification with, and involvement in the organization: Meyer and Allen, 1991). This type of organizational commitment is considered a beneficial psychological state that correlates with positive outcomes both for companies and employees (Meyer and Maltin, 2010). Following a research design based on the communicative methodology (Gomez, 2021), the findings of this study draw from the dialogues with 29 workers of eight MC cooperatives located in the Basque Country, in Spain. The qualitative nature of this study allows complementing, albeit modestly, the vast and fruitful quantitative research on organizational commitment of the past decades.

The results of our analysis suggest that participation in management and ownership contributes to developing affective organizational commitment in at least three ways. First, in relation to participation in management, evidence was found showing that the worker-members interviewed feel identified with the members that form part of the governing bodies of their cooperatives, particularly of the Governing and Social Councils. According to the testimonies, identification originates from the fact that worker-members themselves nominate (for the Governing Council) and choose (for the Social Council) colleagues who they rely on and trust for those representative positions. This democratic process generates a feeling that trustworthy people are in charge of making the most important decisions for the cooperative, such as appointing and monitoring the management staff. While previous research had observed that cooperative values as a democratic organization and participatory management had become decoupled from worker’s daily activity (Heras-Saizarbitoria, 2014), our findings do not seem to reinforce that claim. Instead, our research suggests that a grassroots democracy underlies the process by which representatives are appointed for the governing bodies in MC cooperatives, at least in the cases documented in this study. Further research could contribute to elucidating the extent to which democratic organization and practices are present in the day-to-day life of this and other cooperatives at MC.

Second, also in relation to participation in management, our evidence shows that the involvement of worker-members in governing bodies like the Governing Council and the Social Council enhances their understanding of the business and the operation of the cooperative. According to the interviewees, this happens because the members of the governing bodies must receive specialized training that enables them to understand the information that will be at their disposal and make adequate, informed decisions. Additionally, members of these bodies frequently interact with the management staff, who share information about the status of the cooperative with them. This enhanced understanding of the business by those members seems to increase the motivation, engagement, and empathy toward other members in the same representative positions. Previous research had already shown that involvement in strategic management (Hidalgo-Fernández et al., 2020), decentralization and participative decision-making (Agirre et al., 2014) all have a positive relationship with workers’ organizational commitment in worker cooperatives. However, our research suggests that one factor that could explain such positive relationship is the intensive learning process that takes place as worker-members actively participate in management, for instance in the governing bodies of the cooperative.

Finally, in relation to participation in ownership, our evidence suggests that worker-members develop an emotional attachment to their cooperatives as they acknowledge themselves as co-owners of the companies and part of collective business efforts. The worker-members interviewed clearly expressed that this emotional attachment increased their personal commitment toward their cooperatives, as they felt more motivated to work hard and take on greater responsibilities for the sake of the business, particularly during difficult times. This finding reinforces the argument of previous research that reported that increased job demands and workloads in cooperatives do not reduce organizational commitment of their members (Park, 2018), although our study suggests that a factor that could explain those observations is the emotional attachment that participating in ownership generates. Besides, previous research on psychological ownership had demonstrated positive links between psychological ownership for the organization and employee attitudes like organizational commitment (Van Dyne and Pierce, 2004), which seems to be clearly reinforced by the findings of our research. Future research could further explore the relationship between democratic participation in MC cooperatives, both in management and ownership, with the development of psychological ownership (i.e., the feeling of possessiveness and of being psychologically tied to an organization: Pierce et al., 2001) of their workers.

Overall, in line with the findings of Weber et al. (2020) that demonstrate a positive effect of organizational democracy (i.e., SAEP, EO, and IPD) over value-based commitment, our results show that both participation in management and participation in ownership in MC cooperatives do influence the development of affective commitment of its worker-members (and perhaps also of temporary workers as suggested by some of the testimonies presented). On the one hand, these insights may contribute to inform those worker cooperatives and other democratic organizations that seek to attract and retain human talent. By means of promoting democratic participation of workers in management and ownership, as analyzed in this study, they could potentially increase the affective organizational commitment of their workers. On the other hand, these findings may also contribute to inform the actions of worker cooperatives that deal with the challenge of developing positive types of organizational commitment in their subsidiaries, as they may decide to advance with greater determination in the transfer of these types of democratic participation.

Nevertheless, we must also acknowledge at least three limitations that are inherent to our study. First, due to the size of our sample, it is not possible to extrapolate our findings to all the cooperatives that belong to MC. Instead, the results of our research represent only the feelings of the people interviewed, which are nonetheless valuable to advance in the understanding of the reality of this cooperative group. Second, while the exclusive focus on affective organizational commitment showcased the potential of the democratic participation of workers to generate higher levels of organizational commitment, broadening the focus of analysis to other types of organizational commitment (i.e., continuance and normative) could potentially allow the identification of contradictions and/or exclusionary elements that this research did not identify. Finally, while it would be interesting to contrast the interpretations of the interviewees with quantitative measurements of organizational commitment in their cooperatives, this study did not carry out such statistical analyzes. Future studies could adopt a mixed approach that incorporates into the analysis both the voices of workers and more traditional quantitative measurements in this field of study.

## Data Availability Statement

The data analyzed in this study is subject to the following licenses/restrictions: The information stored on the CREA server can only be consulted upon request when the person responsible for the server and the data of the CREA center is asked. Requests to access these datasets should be directed to Community of Research on Excellence for All (CREA) crea@ub.edu.

## Ethics Statement

The studies involving human participants were reviewed and approved by the Ethics Committee of the Community of Research on Excellence for All (CREA) with the number 20211031. The patients/participants provided their written informed consent to participate in this study.

## Author Contributions

RF: conceptualization, funding acquisition, and supervision. AR-O and AB-F: investigation. MJ: methodology, visualization, and preparation. AR-O: writing – original draft. RF and AB-F: writing – review. All authors contributed to the article and approved the submitted version.

## Conflict of Interest

The authors declare that the research was conducted in the absence of any commercial or financial relationships that could be construed as a potential conflict of interest.

## Publisher’s Note

All claims expressed in this article are solely those of the authors and do not necessarily represent those of their affiliated organizations, or those of the publisher, the editors and the reviewers. Any product that may be evaluated in this article, or claim that may be made by its manufacturer, is not guaranteed or endorsed by the publisher.
